# Oocyte Retrieval in Asymptomatic Patients Positive for SARS-CoV-2

**DOI:** 10.1155/2022/3107747

**Published:** 2022-08-12

**Authors:** Allison Akers, Erika P. New, Shayne Plosker, Celso P. Silva, Rachel Sprague, Anthony N. Imudia

**Affiliations:** ^1^Department of Obstetrics and Gynecology, University of South Florida, Morsani College of Medicine, 2 Tampa General Circle, 6th Floor, Tampa, FL 33606, USA; ^2^Shady Grove Fertility Tampa Bay, 5016 W Cypress St Suite 302, Tampa, FL 33607, USA

## Abstract

**Objective:**

To report two cases of oocyte retrieval performed in asymptomatic SARS-CoV-2-positive patients.

**Design:**

Case report. *Setting*. Outpatient private practice infertility center. *Patients*. A 28-year-old woman at risk for OHSS who took her trigger injection prior to testing positive for SARS-CoV-2 and a 19-year-old oncofertility patient who tested positive prior to retrieval due to a family exposure. Both patients were asymptomatic. *Main Outcome Measures*. Cycle outcomes, patient safety, and staff safety.

**Results:**

Both patients underwent successful oocyte retrieval procedures without developing symptoms or complications from COVID-19. No staff members that cared for these patients developed symptoms of COVID-19.

**Conclusion:**

Worsening fertility outcomes and potential for psychological and financial burdens to the patient must be balanced with risk of perioperative complications in patients testing positive for SARS-CoV-2. As we continue to provide fertility care in a world with COVID-19, appropriate risk mitigation strategies should be implemented to minimize exposure to SARS-CoV-2.

## 1. Introduction

Amid the ever-changing COVID-19 pandemic, many fertility clinics have fully resumed patient care, however with necessary adjustments to reduce the spread of COVID-19 and optimize patient safety. At the beginning of the pandemic, there were many ovulation induction, intrauterine insemination (IUI), and in vitro fertilization (IVF) cycles cancelled after the American Society for Reproductive Medicine (ASRM) released practice recommendations to suspend initiation of all new treatment cycles on March 17, 2020 [1]. This abrupt suspension of therapy resulted in significant interruption to fertility care during the early months of the COVID-19 pandemic. In a survey of 1,808 women in the US who primarily self-identified as having infertility, only 13.7% of respondents indicated that their treatment cycles had continued as planned following the release of initial ASRM guidelines, demonstrating the pandemic's vast disruption to timely fertility care [2]. Now that infertility clinics are back to caring for IVF patients with infertility, there have been cycle cancellations due to patients' presence of COVID-19 symptoms or confirmed infection detected by SARS-CoV-2 polymerase chain reaction testing.

Most medical institutions have implemented universal preoperative SARS-CoV-2 testing prior to performing elective, outpatient surgeries. The goal is to identify patients who may be in the early incubation period of COVID-19 infection and may have increased morbidity if undergoing intubation during this time [3]. Specific to infertility practices, COVID-19 screening and/or SARS-CoV-2 testing has become the standard prior to providing patient care and especially performing surgical procedures. In a large volume academic IVF program in New York City, universal SARS-CoV-2 testing of infertility patients within five days of oocyte retrieval found an overall positivity rate of 0.4%. All six patients who tested positive prior to retrieval had their cycles cancelled. No complications, such as ovarian hyperstimulation syndrome (OHSS), occurred in these six women [4].

The impact of COVID-19 on treatment cancellations has lasting ramifications. In a recent survey of patients who had a cycle cancelled due to the COVID-19 pandemic, 85% of respondents found it to be moderately to extremely upsetting with 22% rating it to be equivalent to the loss of a child [5]. Patients of advanced maternal age and with a previous IVF attempt were particularly at risk for anxiety and depression associated with the pandemic's impact on treatment [6]. COVID-19 and treatment cancellations have also exacerbated the financial strain associated with assisted reproductive technology (ART). Average ART treatment costs have been estimated at greater than $15,000 per fresh cycle and $3,000 per frozen cycle [7]. For many patients, the level of affordability of ART is a key determinant to access and utilization of fertility treatments [8, 9]. Cancellations of treatment during the COVID-19 pandemic represent a substantial financial barrier for patients and pose a potential obstacle for continued treatment. Additionally, public health efforts to slow the spread of COVID-19 have lead millions of Americans to lose jobs and insurance coverage [10]. While the exact impact on treatment continuation is unknown, this may lead to further financial barriers to treatment. The ASRM acknowledges that delaying ART may have permanent negative consequences to treatment outcome, mental health, and access to care [1]. ART cancellations result in financial and psychologic stress to patients and may violate the right to reproduce, reflecting underlying structural views that marginalize reproductive health [11].

Our infertility clinic has not implemented universal testing for patients. Rather, we have implemented a triage and screening approach for COVID-19 symptoms as well as universal precautions for COVID-19. All patients are required to wear masks and must undergo screening with a temperature scan and completion of a questionnaire prior to being admitted to the clinic. If there is a positive screen or elevated temperature, then the case is discussed with the patient's physician, and COVID-19 testing is recommended prior to patient care. Recently, we had two cases where patients who voluntarily sought out COVID-19 testing a few days prior to oocyte retrieval tested positive although they were asymptomatic for COVID-19. Both underwent oocyte retrieval. To our knowledge, there have been no case reports in the literature describing outcomes of retrieval in COVID-19-positive patients. Informed consent was obtained from both patients for this case report. We will discuss these challenging cases as well as the decision-making process that can be employed when caring for these patients.

## 2. Case One: OHSS Risk in a Patient Who Triggered before Testing Positive

A 28-year-old G0 female with primary infertility, dysmenorrhea, and obesity (BMI 36) presented to our Reproductive Endocrinology and Infertility practice desiring to conceive. On infertility evaluation, she was found to have bilateral endometriomas (3.0 cm and 1.3 cm) and recurrent endometrial polyps which were removed by hysteroscopic polypectomy. Ovarian reserve testing revealed an antral follicle count (AFC) of 12 and anti-müllerian hormone (AMH) of 4.55. Semen analysis of the male partner revealed teratozoospermia (concentration 128.2 million/ml, motility 65.6%, rigid morphology 1%). The couple elected to proceed with IVF including preimplantation genetic testing for aneuploidy (PGT-A). After pretreatment with oral contraceptive pills, an antagonist protocol was utilized. After eleven days of stimulation, the patient had a peak estradiol of 5,801 pg/ml, a progesterone level of 1.18 ng/ml, 32 follicles imaged on ultrasound, and 19 follicles > 14 mm diameter in size. She was triggered with a single dose of Lupron 4 mg. On the evening she took her trigger injection, the patient's partner became symptomatic and tested positive for COVID-19. Both the patient and her partner were unvaccinated against COVID-19. The day after her trigger, the patient underwent rapid testing for SARS-CoV-2 and tested positive. She was asymptomatic. The patient was instructed not to present to the clinic the morning after Lupron administration for posttrigger labs in the setting of positive SARS-CoV-2 testing, and the option of cycle cancellation was reviewed. She was counseled on the risks of COVID-19 infection in the setting of a surgical procedure. The decision was made to proceed with oocyte retrieval 36 hours after trigger, with additional precautions taken to minimize risk of infection to staff and other patients. Based on the timing of her trigger, the patient's retrieval occurred as the last retrieval of the day. The patient was asked to wait in her car until specifically instructed to enter the facility so that all other patients could be discharged from the PACU prior to her entering the building. Her partner was instructed to produce his semen specimen at home rather than in the practice collection room. Only fully vaccinated, essential staff necessary for patient care were present in the preoperative, operating room (OR), and PACU area. All staff and the patient each wore an N95 mask. During the oocyte retrieval, total IV anesthesia was utilized (TIVA) in our ambulatory surgical center outpatient OR without complications. During the retrieval, 19 oocytes were retrieved, 17 of which were mature and underwent ICSI, resulting in 10 blastocysts. PGT-A testing revealed 8 euploid blastocysts. The patient remained on the GnRH antagonist (Cetrotide) 0.25 mg daily for 7 days postoocyte retrieval as a precautionary measure. The patient did well postoperatively with no evidence of OHSS and no symptoms of COVID-19 infection. No additional SARS-CoV-2 testing was performed as per the September 2021 CDC guidance recommending that patients who remain asymptomatic should not be retested within 90 days [12]. Two months after oocyte retrieval, the patient underwent frozen embryo transfer of a single euploid blastocyst. Her initial posttransfer hCG level was 2,916 mIU/ml with an appropriate increase 48 hours later to 6,817 mIU/ml. She underwent obstetrical ultrasound confirming a viable, singleton intrauterine gestation. She transitioned medical care to her obstetrician gynecologist at 8 weeks gestation for her ongoing pregnancy.

## 3. Case Two: Oncofertility Patient Who Tested Positive prior to Retrieval due to Exposure

A 19-year-old G0 female presented for fertility preservation via oocyte cryopreservation in the setting of a recent diagnosis of acute promyelocytic leukemia, having completed one round of chemotherapy treatment (arsenic trioxide/ATO and all-trans-retinoic acid/ATRA) with plans for further consolidation chemotherapy using the same ATO/ATRA regimen. The patient had no other significant past medical history. She had a hormonal IUD in place for the past 10 months. A random start antagonist protocol was initiated. On day 10 of stimulation, the patient did not present to the clinic for her scheduled appointment. When contacted by phone, she reported that she had multiple family members who had tested positive for COVID-19; therefore, she obtained rapid testing, which was positive for SARS-CoV-2. The patient was asymptomatic. She continued her scheduled dose of stimulation medications, and the following day, she underwent confirmatory SARS-CoV-2 PCR testing which also returned positive. She was counseled on the risks of proceeding with oocyte retrieval, and she desired to proceed. The patient was then triggered that evening (day 11 of stimulation) with hCG 5,000 IU. The decision to use an hCG trigger instead of a GnRH agonist trigger was to reduce the risk of a failed trigger, especially as a failed GnRH agonist trigger would not be identified until day of retrieval due to not obtaining posttrigger labs. Oocyte retrieval was performed 36 hours later. Due to the patient not presenting to clinic, the last estradiol level and ultrasound data we have was on stimulation day 9, with a peak estradiol level of 3,067 pg/ml, progesterone of 0.77 ng/ml, and 37 follicles imaged sonographically, with 16 follicles > 14 mm in diameter. She underwent an uncomplicated oocyte retrieval obtaining 35 oocytes. Twenty-two mature oocytes were vitrified. The same infectious disease precautions were used as for case 1. The patient did well postoperatively without symptoms and was able to continue with her planned chemotherapy treatment five days after oocyte retrieval.

## 4. Discussion

In the first case, the patient had a known COVID-19 exposure, and she subsequently tested positive for SARS-CoV-2 the day after taking her Lupron trigger. She was asymptomatic. The risks of cancelling the cycle versus proceeding with retrieval were thoroughly considered by several physicians in the practice, as well as with risk management. This patient had a high peak estradiol of 5,801 pg/ml and had already taken the Lupron trigger when testing positive. As such, she was at risk for developing OHSS. Current literature describing the risks of OHSS in COVID-19-positive patients is limited but describes more severe outcomes. A case report described a patient with mild COVID-19 infection who developed significant bilateral pleural effusions requiring bilateral thoracentesis with only minimal abdominal ascites [13]. Isolated pleural effusions without significant ascites are a rare presentation of OHSS, suggesting recent COVID-19 infection may result in increased fluid accumulation preferentially to the lungs during OHSS. Additionally, thromboembolic events have been described in patients with COVID-19 and are associated with poor prognosis in COVID-19 infections. It is well known that ART procedures are associated with thromboembolic complications, most of which are reported in the context of OHSS [14]. We did not prescribe an anticoagulant postoocyte retrieval, carefully weighing the risk of post-op bleeding *vs.* the risk of thromboembolism in our asymptomatic, ambulatory patient. While knowledge of outcomes in patients with COVID-19 infection and OHSS is limited, current data suggests that physiologic changes seen with fertility treatment may be exacerbated by common pathophysiological aspects of COVID-19 viral infection. In our case, we believed that without retrieval, this patient would have been at risk for developing OHSS that would have been further complicated and potentially worsened by her COVID-19 infection.

Despite the dangers associated with cancelling the patient's cycle, proceeding with retrieval was not without risk to both the patient and medical staff who cared for her. In one international, multicenter cohort study, postoperative pulmonary complications occurred in half of patients with perioperative COVID-19 infection and were associated with high mortality [15]. However, increased 30-day mortality was associated with male sex, age 70 years or older, malignant diagnoses, emergency versus elective surgery, and major versus minor surgery, none of which apply to our patient. Another study evaluating surgical outcomes of patients with COVID-19 found similar results with 30-day mortality significantly higher in COVID-19-positive patients compared to controls [16]. However, this study excluded gynecological and minor surgical procedures, limiting its relevance to our patient's case. Nonetheless, the American Society of Anesthesiologists (ASA) acknowledges this current knowledge as the basis for extrapolated wait times between COVID-19 diagnosis and surgery. The ASA suggests four weeks from the diagnosis of COVID-19 until elective surgery for asymptomatic patients but highlights that these are not definitive and should factor in individualized preoperative risk assessment [17].

The ASA recommendations for timing of elective surgery after recovery from COVID-19 prompt the question of what truly constitutes “elective” procedures. During the pandemic, elective procedures have typically been defined based on implications for physical health and survival [11]. However, this deprioritizes interventions whose benefits extend beyond just survival, including ART. An alternative ethical framework for defining “elective” procedures in the time of resource scarcity proposes that delay in care is not elective if it comes with intolerable costs, violates patient autonomy, or deferral results in permanent injury [18]. In this case, cancelling the patient cycle would not only have physical implications with increased risk of OHSS but would also result in financial and emotional burdens for the patient. As such, one could argue that under this ethical framework, oocyte retrieval for our patient was far from elective.

Another consideration for this patient's care concerns her male partner who not only tested positive for SARS-CoV-2 but was also symptomatic. COVID-19 infection can have a negative impact on semen analysis parameters, with potential long-term implications such as germ cell depletion and hypogonadism [19]. Semen analyses from men who had recovered from COVID-19 showed abnormalities such as azoospermia and oligospermia compared to healthy controls, requiring a median of three months to see improvement in semen parameters [19]. However, most studies have found no evidence of SARS-CoV-2 viral RNA in semen of male patients with COVID-19 infection [20]. We chose to proceed with ICSI the day of the retrieval for these reasons; however, another option would have been to freeze oocytes and inseminate them later after her partner had fully recovered from his infection.

The implication of potential SARS-CoV-2 infection on embryo development and risk during trophectoderm biopsy for PGT-A was also considered. Studies suggest that *ACE2* and *TMPRSS2*, both of which are critical in SARS-CoV-2 entry into cells, are expressed in some trophoblast, syncytiotrophoblast, and hypoblast cells, suggesting that embryos may be susceptible to COVID-19 infection [21–23]. Hong et al. showed susceptibility of endoderm and ectoderm cells to infection in human lung cells directly inoculated with SARS-CoV-2 virus in a laboratory setting [23]. In the clinical setting however, there has been no detectable viral RNA for SARS-CoV-2 found in the follicular fluid, cumulus cells, or endometrial tissue samples from women testing positive for SARS-CoV-2 48 hours prior to oocyte retrieval [24]. In sixteen COVID-19-positive patients undergoing oocyte retrieval and embryo culture with PGT-A biopsy if indicated, there were similar fertilization rates, embryo quality, and development as well as frozen embryo transfer outcomes [24]. While further studies are needed, there is reasonable data to suggest that proceeding ICSI, embryo culture, and biopsy in this setting was a reasonable choice for our patient.

It is important to highlight that this patient was young, a good responder, and, cost aside, would likely be able to undergo another cycle later without diminished changes of future pregnancies. In patients with diminished ovarian reserve, a delay in initiating IVF treatment up to 180 days from the initial visit does not affect pregnancy outcomes, and our patient would also likely have similar pregnancy outcomes should her cycle have been cancelled [25]. However, we believed that risks associated with cancelling the cycle for this patient outweighed potential perioperative complications and similar pregnancy outcomes. As such, we proceeded with successful oocyte retrieval.

In the second case, the patient tested positive for COVID-19 on day 10 of stimulation. She also was asymptomatic. The risks of proceeding with retrieval were again compared to the benefits of cancelling the cycle. In contrast to the first case, this patient who would be proceeding with gonadotoxic chemotherapy would likely not have similar fertility outcomes should her cycle be cancelled. Patients who have a cancer diagnosis and desire future fertility are a unique and vulnerable population that must receive special consideration, especially during the COVID-19 pandemic. Leukemia, breast cancer, endometrial cancer, and cervical cancer are just a few malignancies that impact reproductive age women, highlighting the importance of counseling and offering timely fertility preservation options for those who have not yet completed childbearing [26, 27]. The very treatments that help cure these devastating diseases have a significant impact on future fertility. For example, chemotherapeutic agents can cause permanent damage to ovarian primary follicles causing premature ovarian failure [26]. In some diagnoses such as endometrial cancer, there has been an important focus on fertility sparing treatment for select women with low-stage disease; however for those in which fertility sparing treatment is contraindicated, hysterectomy with or without additional chemotherapy and radiation significantly harms future fertility [27, 28]. On a population level, the need for fertility preservation services increases as more women delay childbearing. This delay increases the chance that a cancer diagnosis is made prior to a patient starting or completing their family building [28]. Providing oncofertility patients with proper counseling and access to timely fertility preservation services is always a challenge but has become more so during the COVID-19 pandemic due to decreased access to elective procedures and increased patient anxiety about risk of infection during treatment, potentially further delaying cancer treatment [26]. Our patient was fortunate to be referred for fertility preservation prior to proceeding with further chemotherapy treatment, as is the recommendation for patients receiving gonadotoxic treatment [29].

Infertility is a well-known consequence of combination chemotherapy given for leukemia, although the reported rates differ based on various factors including chemotherapeutic protocol and age at treatment [30]. Not proceeding with trigger and egg retrieval at this late stage of stimulation in this case could possibly increase the risk of OHSS and ovarian torsion which could delay the continuation of planned treatment for leukemia. The Ethics Committee of the ASRM highlights that all available options for fertility preservation should be offered to patients receiving gonadotoxic therapies [29]. In our patient, cancelling her cycle could have resulted in decreased future reproductive potential. In addition, as the initial treatment plan was for oocyte vitrification, this plan did not require any adjustment based on COVID-19 infection status.

In the setting of COVID-19 infection, this patient had similar risks of perioperative complications compared to the first case. While some studies show that surgery for malignant disease was associated with worsening perioperative outcomes in the setting of COVID-19 infection, it is unclear whether this patient's leukemia itself was an independent risk factor for complications in the setting of COVID-19 infection and surgery [15]. Furthermore, cancelling this patient's cycle would have also reduced exposure for staff and other patients. However, we believed that the worsening fertility outcomes and potential for psychological and financial burdens to the patient outweighed theoretical perioperative complications. Thus, we chose to proceed with the retrieval.

## 5. Conclusion

As the pandemic continues, new practice guidelines will be necessary to optimize ART care for patients with or exposed to COVID-19. Our practice was able to safely perform oocyte retrieval on two asymptomatic patients who tested positive for COVID-19 by minimizing in-person exposure prior to retrieval, reducing staff to essential, vaccinated personnel, and utilizing appropriate PPE including N95 masks for all staff involved with direct patient care based on the ASA recommendations for COVID-19 precautions [17]. These as well as other important considerations are listed in Table 1. TIVA was utilized in both cases. However, if intubation is needed, it is recommended to perform procedures in airborne infection isolation rooms due to negative pressure relative to surrounding areas. While the ASA recommends 4 weeks in between asymptomatic COVID-19 diagnosis and elective surgery, individualized assessment is necessary in determining the benefit/risk ratio of delaying surgery.

These cases demonstrate the challenges in determining whether to proceed with fertility treatment in COVID-19-positive patients. More discussion is necessary on how to proceed should a patient test positive for COVID-19 and is asymptomatic during ART. Clinicians must weigh the risks of OHSS and potential for decreasing fertility with perioperative complications associated with COVID-19 infection. Additionally, the psychological and financial burdens associated with cycle cancellation and patient autonomy must also be factored into the decision. As we continue to provide fertility care in a world with COVID-19, an algorithm may be necessary to help clinicians proceed with these difficult decisions.

We recommend cycle cancellation, per ASA guidelines, when COVID-19 infection is diagnosed in the early days of stimulation. However, we believe that cases must be individualized to balance risk/benefit ratios for each patient. If it is necessary to proceed with a procedure, appropriate risk mitigation strategies should be implemented to minimize COVID-19 exposure.

## Figures and Tables

**Table 1:** Considerations for patients with asymptomatic COVID-19 infection undergoing ovarian stimulation.

*Pretreatment*
Have a comprehensive practice policy in place for screening for COVID-19 prior to patient care.
Encourage practice personnel involved with patient care to become fully vaccinated against COVID-19.
Encourage patients and their partners to become fully vaccinated against COVID-19.
Ensure that all staff members, including contracted personnel, have undergone proper N95 fit testing.
*During treatment*
Advise symptomatic patients, or patients exposed to a known COVID-19-positive person, to inform the clinic and to undergo COVID-19 testing regardless of vaccination status.
*If a patient tests positive for COVID-19*
Patient symptoms should be ascertained.
If the patient is symptomatic, discuss cycle cancellation.
If the patient is asymptomatic, the decision to proceed or not to proceed with oocyte retrieval should be made based on practice policy, in consultation with colleagues and the anesthesia team.
*Preretrieval and day of retrieval*
Limit contact between the patient and staff as well as other patients by (i) not performing posttrigger labs the day before the retrieval (ii) collecting semen specimen at home and bringing to the clinic (iii) performing oocyte vitrification or embryo freeze-all to allow the patient to fully recover if symptoms develop postretrieval and further reduce exposure
Staff and patient to wear proper PPE including N95 masks.
Avoid front desk check-in by the infected patient. Have her proceed directly to the oocyte retrieval area.
If possible, schedule the retrieval such that all prior patients have exited the pre- and postoperative recovery areas prior to arrival of the infected patient.
*Postretrieval*
Clean all patient contact areas with disinfectant products that have been proven effective against COVID-19 based on the Environmental Protection Agency's List N of disinfectants [31].
Mitigate OHSS risk.
Patient to continue to use N95 mask when in public.
Frequent follow-up by phone with the patient to assess for symptoms and need for further evaluation or treatment.

## Data Availability

No data were used.
